# Serum cotinine, lipid accumulation product, and the mediating role of HOMA-IR among US adults: NHANES 2013–2018

**DOI:** 10.18332/tid/226322

**Published:** 2026-10-21

**Authors:** Chi Zhuang, Dingxiang Hu, Lejin Hong, Jing Chen, Zhi Chen

**Affiliations:** 1 The School of Basic Medical Sciences, Fujian Medical UniversityFuzhouChina; 2 Department of Rehabilitation TherapyJiangsu Second Rongjun Hospital (Jiangsu Rongjun Hospital)WuxiChina; 3 Department of OrthopedicsZhangpu HospitalZhangzhouChina; 4 Department of OphthalmologyFuzhou University Affiliated Provincial HospitalFuzhouChina; 5 Department of Orthopedics Sports Injury DivisionFujian Medical University Union HospitalFuzhouChina

**Keywords:** HOMA-IR, serum cotinine, lipid accumulation product, insulin resistance, nicotine exposure

## Abstract

**Introduction:**

The lipid accumulation product (LAP) is a practical indicator of abdominal lipid accumulation and triglyceride-related metabolic burden, and has been linked to cardiometabolic abnormalities. Smoking and nicotine exposure may influence lipid metabolism, fat distribution, and insulin resistance, all of which could contribute to variation in LAP. Serum cotinine, a stable metabolite of nicotine, is an objective biomarker of recent nicotine intake. However, evidence on the association between serum cotinine and LAP and the potential mediating role of HOMA-IR remains limited. This study aimed to examine these associations using NHANES 2013–2018 data.

**Methods:**

Data from NHANES 2013–2018 were analyzed, including 6409 participants with complete data on serum cotinine, waist circumference, triglycerides, fasting glucose, and insulin. LAP was computed with sex-specific formulas. Weighted linear regression models were used to evaluate associations of cotinine and the homeostasis model assessment of insulin resistance (HOMA-IR) with LAP. Mediation analysis then assessed the extent to which HOMA-IR statistically explained the cotinine–LAP association.

**Results:**

Participants with higher LAP had elevated cotinine and HOMA-IR. After full covariate adjustment, cotinine was positively associated with LAP (β=0.019; 95% CI: 0.011–0.028; p<0.001). Compared with the lowest cotinine tertile, the highest tertile corresponded to a 3.142-unit increase in LAP (p=0.016), with a significant trend across tertiles (p-trend=0.017). HOMA-IR also showed a positive association with LAP (β=1.345; p<0.001). In mediation analysis, HOMA-IR explained 7.4% of the cotinine–LAP association (indirect effect=0.003).

**Conclusions:**

Serum cotinine, a biomarker of recent nicotine intake, was positively associated with LAP in this cross-sectional NHANES analysis. HOMA-IR statistically explained only a small proportion of this association. These findings should be interpreted as associations rather than evidence of causality.

## Introduction

Metabolic disturbances related to excessive adipose accumulation, particularly abdominal lipid deposition, have become a major contributor to the burden of cardiometabolic diseases^1^. Central adiposity is closely associated with dyslipidemia, hypertension, type 2 diabetes, insulin resistance, and cardiovascular disease, suggesting that adipose tissue distribution and metabolic activity may provide more informative insight into metabolic risk than body size alone^2^. Although body mass index (BMI) is widely used, it does not reflect regional fat distribution or triglyceride-related metabolic burden. LAP, a sex-specific index calculated from waist circumference and fasting triglycerides, may better capture abdominal lipid accumulation and has been associated with cardiometabolic outcomes, including metabolic syndrome, type 2 diabetes, non-alcoholic fatty liver disease, and cardiovascular risk^3-8^. Because LAP reflects both abdominal adiposity and triglyceride metabolism, exposures that influence lipid handling, fat distribution, or insulin sensitivity may be relevant to its variation. Smoking and nicotine exposure are of particular interest in this context because of their established disease burden and potential metabolic effects^9,10^. Beyond their established effects on cardiovascular, respiratory, and cancer-related outcomes, smoking and nicotine exposure have increasingly been linked to metabolic health^10^. Review-level evidence suggests that current smoking may increase susceptibility to metabolic syndrome, while both active and passive smoking have been implicated in the later development of type 2 diabetes^11,12^. Mechanistic and epidemiological evidence indicates that smoking and nicotine exposure may contribute to dyslipidemia, insulin resistance, and other cardiometabolic disturbances by affecting lipid metabolism, oxidative stress, and insulin signaling pathways^10^. Cotinine is a major metabolite of nicotine and has a longer half-life than nicotine, making serum cotinine a widely used biomarker of recent nicotine intake^13^. Compared with self-reported smoking status, serum cotinine can reduce exposure misclassification caused by under-reporting or inaccurate reporting^14^. Although smoking and tobacco product use have been studied in relation to multiple metabolic outcomes, their specific relationship with LAP has not been adequately investigated. A study of Korean adults reported that both single and dual tobacco product use were associated with higher LAP levels; however, that study was mainly based on behavioral categories of tobacco use and did not directly assess nicotine exposure using serum cotinine^15^. Conversely, studies using cotinine as an exposure biomarker have tended to focus on lipid ratios, atherogenic lipid indices, or metabolic syndrome, rather than LAP as the primary outcome^16,17^. Because LAP incorporates both waist circumference and fasting triglyceride concentration, it may provide a more targeted representation of abdominal lipid overaccumulation^4^. Therefore, examining serum cotinine in relation to LAP may help clarify whether recent nicotine intake is associated with lipid accumulation-related metabolic abnormalities^10,14^.

Insulin resistance may also be involved in the association between serum cotinine and LAP. Nicotine exposure has been reported to interfere with insulin signaling and glucose metabolic regulation, and these disturbances may contribute to broader cardiometabolic dysfunction^10,18^. In insulin-resistant states, the antilipolytic effect of insulin on adipose tissue is weakened, leading to increased free fatty acid flux to the liver, enhanced hepatic triglyceride synthesis, and greater secretion of triglyceride-rich lipoproteins^19^. These metabolic processes are closely related to the two components of LAP, namely waist circumference and fasting triglycerides^4^. HOMA-IR, calculated from fasting glucose and fasting insulin concentrations, is widely used in epidemiological studies as an indirect measure of insulin resistance^20^. Existing evidence also indicates that LAP and other lipid-related indices are associated with insulin resistance and systemic inflammation^21,22^.

Previous studies have examined smoking behavior or tobacco product use in relation to LAP, whereas studies using serum cotinine have mainly focused on conventional lipid indices or metabolic syndrome^15-17^. To address this gap, the present analysis used NHANES 2013–2018 data to examine the association between serum cotinine and LAP and to evaluate whether HOMA-IR statistically mediates this association.

## Methods

### Data source

This study was a secondary analysis of cross-sectional data from NHANES 2013–2018. NHANES is conducted by the National Center for Health Statistics and uses a stratified multistage sampling design to represent the non-institutionalized US population. Data are collected through interviews, physical examinations, and laboratory tests. The survey was approved by the National Center for Health Statistics Ethics Review Board, and all participants provided informed consent.

### Study population

This investigation utilized data from three NHANES cycles (2013–2018). Participants with complete records for cotinine, as well as necessary data to compute LAP (waist circumference and triglycerides) and HOMA-IR (fasting insulin and fasting plasma glucose), were included. Exclusion criteria were: missing data for waist circumference (n=7856), triglycerides (n=14439), fasting glucose (n=17), insulin (n=89), pregnancy (n=52), cancer diagnosis (n=537), and cotinine (n=1). The detailed screening procedure is presented in Figure 1.

**Figure 1:**
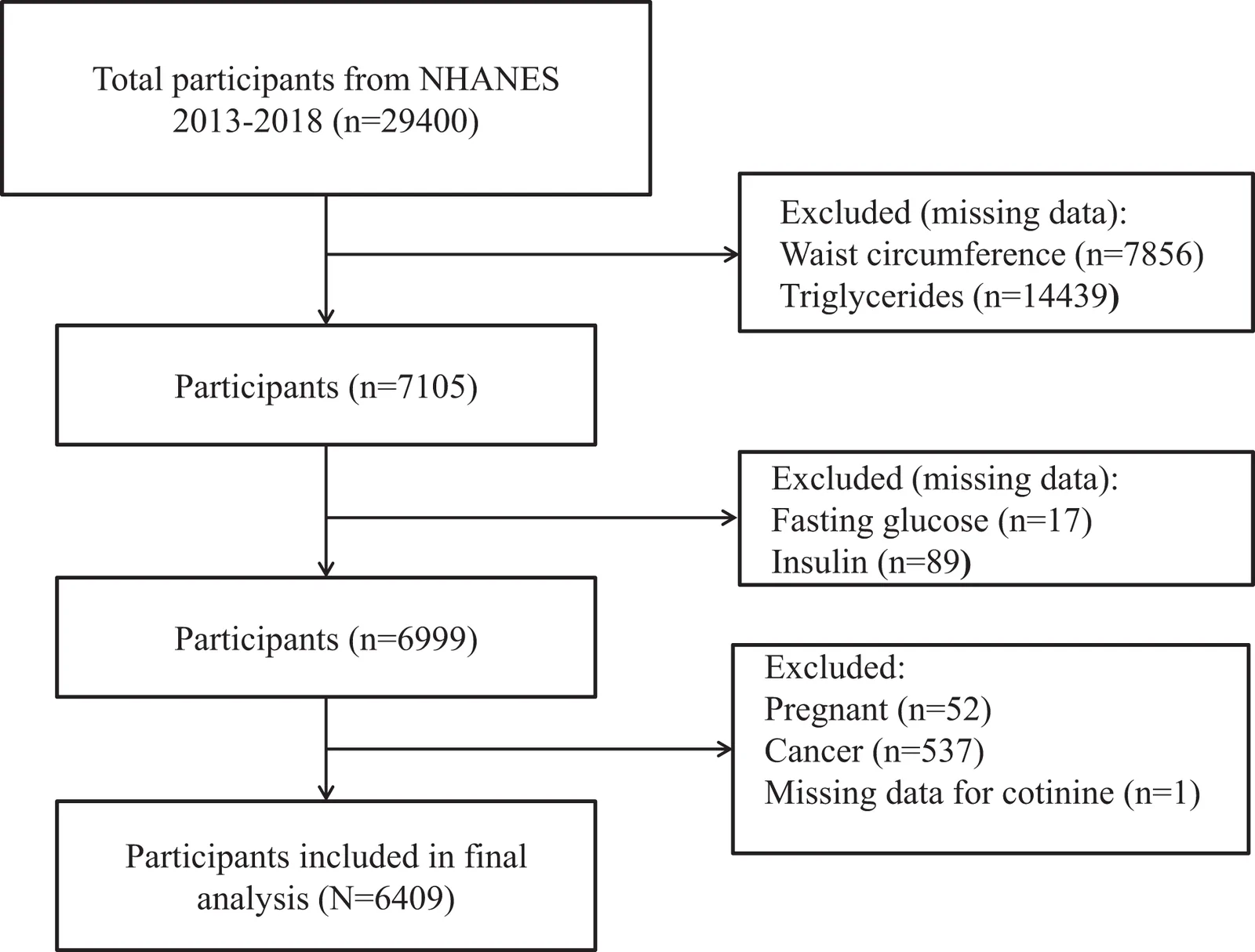
Flowchart of participant selection from NHANES 2013–2018, a US nationally representative cross-sectional survey (final analytical sample, N=6409)

### Study variables

#### Serum cotinine

Serum cotinine concentrations were quantified in NHANES laboratory testing by isotope-dilution high-performance liquid chromatography coupled with atmospheric-pressure chemical ionization tandem mass spectrometry (ID HPLC–APCI MS/MS). Serum cotinine was entered as a continuous variable on its original scale without logarithmic transformation.

#### HOMA-IR

The HOMA-IR was calculated^20^ as: HOMA-IR = fasting insulin (μIU/mL) × fasting plasma glucose (mmol/L)/22.5. Plasma glucose and fasting insulin were determined with an immunoenzymometric method on a TOSOH AIA-900 chemistry analyzer.

#### LAP

LAP was computed according to sex-specific equations: for males, LAP = [Waist Circumference (WC, cm) - 65] × [Triglycerides (TG, mmol/L)]; for females, LAP = [WC (cm) - 58] × [TG (mmol/L)^23^.

#### Potential covariates

Potential covariates were selected based on prior literature. Demographic variables, including age and sex (male, female), were obtained from demographics data. BMI (kg/m²) was calculated from measured height and weight obtained during the physical examination. Folate-related biomarkers and biochemical parameters were measured from blood specimens using standardized NHANES laboratory protocols. Specifically, 5-methyl-tetrahydrofolate (ng/mL) was assayed by isotope-dilution liquid chromatography–tandem mass spectrometry; serum total folate (ng/mL) was calculated as the sum of individual folate forms; and RBC folate (ng/mL) was determined by a microbiological assay. Albumin (g/dL), globulin (g/dL), AST (U/L), ALT (U/L), uric acid (mg/dL), and LDH (U/L) were measured using a Beckman UniCel^®^ DxC800 Synchron analyzer. Detailed information on laboratory methods, data processing procedures, quality assurance, and analytic notes is provided in the corresponding NHANES documentation^24^.

### Statistical analysis

Statistical processing was performed in R software (version 4.2.2; https://www.r-project.org/) and EmpowerStats (version 2.0; http://www.empowerstats.com/). A two-tailed p<0.05 was considered statistically significant. The complex sampling structure of NHANES was considered by applying the appropriate survey weights, strata, and cluster variables. Baseline characteristics were compared with design-based linear models for continuous variables and weighted chi-squared tests for categorical variables. Univariate linear regression identified factors associated with the LAP. Survey-weighted multivariable linear models examined the relationships among cotinine, HOMA-IR, and LAP.

The R package *mediation* was used to assess whether HOMA-IR partly mediated the relationship between serum cotinine and LAP, and to calculate the corresponding mediation proportion. In this model, cotinine was treated as the exposure, HOMA-IR as the mediator, and LAP as the outcome. Statistical mediation was evaluated based on the estimated indirect effect, direct effect, total effect, and proportion mediated using the R package *mediation*^25^.

## Results

After the exclusion criteria were applied, 6409 participants remained in the analysis. Participants were stratified according to LAP level. Compared with the low LAP group, participants in the high LAP group had higher serum cotinine (47.12 ± 115.50 vs 58.71 ± 126.21 ng/mL), HOMA-IR (1.94 ± 4.11 vs 6.27 ± 10.47), and BMI (22.72 ± 3.65 vs 34.11 ± 7.21 kg/m²), whereas albumin was lower (4.39 ± 0.32 vs 4.20 ± 0.35 g/dL). Full baseline characteristics are shown in Table 1.

**Table 1:** Baseline characteristics according to LAP tertiles among US NHANES 2013–2018 participants (N=6409)

Characteristics	Low LAP tertile (N=2136)	Medium LAP tertile (N=2136)	High LAP tertile (N=2137)	p
LAP (cm × mmol/L)	11.27 ± 5.34	33.36 ± 8.28	99.62 ± 80.67	<0.001
Age (years)	32.43 ± 17.40	45.21 ± 17.90	48.98 ± 16.06	<0.001
Serum cotinine (ng/mL)	47.12 ± 115.50	55.55 ± 130.40	58.71 ± 126.21	0.009
HOMA-IR	1.94 ± 4.11	2.83 ± 3.42	6.27 ± 10.47	<0.001
ALT (U/L)	19.82 ± 15.30	23.23 ± 16.03	28.97 ± 18.25	<0.001
Albumin (g/dL)	4.39 ± 0.32	4.25 ± 0.32	4.20 ± 0.35	<0.001
AST (U/L)	23.76 ± 14.83	23.67 ± 18.89	25.89 ± 18.88	<0.001
Globulin (g/dL)	2.76 ± 0.41	2.81 ± 0.41	2.91 ± 0.44	<0.001
LDH (U/L)	130.15 ± 31.35	131.92 ± 32.39	135.06 ± 32.77	<0.001
Uric acid (mg/dL)	4.86 ± 1.18	5.30 ± 1.24	5.97 ± 1.47	<0.001
RBC folate (ng/mL)	1080.19 ± 401.30	1191.02 ± 522.27	1357.50 ± 617.66	<0.001
Serum total folate (ng/mL)	43.33 ± 20.05	42.22 ± 23.71	43.72 ± 45.76	0.312
5-Methyl-tetrahydrofolate (ng/mL)	41.22 ± 18.98	39.91 ± 22.21	41.25 ± 44.23	0.296
BMI (kg/m²)	22.72 ± 3.65	28.46 ± 5.12	34.11 ± 7.21	<0.001
**Gender**, %				0.236
Male	50.3	48.3	50.7	
Female	49.7	51.7	49.3	

Values are presented as mean ± standard deviation for continuous variables and percentage for categorical variables. LAP: lipid accumulation product. HOMA-IR: homeostasis model assessment of insulin resistance. BMI: body mass index. ALT: alanine aminotransferase. AST: aspartate aminotransferase. LDH: lactate dehydrogenase. RBC: red blood cell. Statistical significance was defined as p<0.05.

### Univariate analyses

In univariate analyses, serum cotinine (β=0.013; 95% CI: 0.001–0.025; p=0.035), HOMA-IR (β=2.482; 95% CI: 2.282–2.683; p<0.001) and BMI (β=3.945; 95% CI: 3.762–4.128; p<0.001) were positively associated with LAP, whereas albumin showed an inverse association (β= -26.326; 95% CI: -30.667 – -21.986; p<0.001). Full univariate results are presented in Table 2.

**Table 2:** Univariate associations with LAP among US NHANES 2013–2018 participants (N=6409)

Variables	Mean ± SD	β (95% CI)	p
**Age** (years)	42.07 ± 20.02	0.619 (0.540–0.699)	<0.001
**Gender**, n (%)			
Male	3161 (49.3)	ref.	
Female	3248 (50.7)	-3.358 (−6.351 – -0.365)	0.028
**Measurements**			
Serum cotinine (ng/mL)	48.67 ± 116.72	0.013 (0.001–0.025)	0.035
HOMA-IR	4.00 ± 7.79	2.482 (2.282–2.683)	<0.001
ALT (U/L)	23.60 ± 17.28	0.679 (0.595–0.762)	<0.001
Albumin (g/dL)	4.26 ± 0.35	-26.326 (−30.667 – -21.986)	<0.001
AST (U/L)	24.47 ± 20.17	0.231 (0.150–0.312)	<0.001
Globulin (g/dL)	2.91 ± 0.44	13.569 (10.564–16.573)	<0.001
LDH (U/L)	134.01 ± 36.54	0.173 (0.128–0.217)	<0.001
Uric acid (mg/dL)	5.39 ± 1.42	11.618 (10.575–12.660)	<0.001
RBC folate (ng/mL)	1151.12 ± 501.36	0.020 (0.017–0.022)	<0.001
Serum total folate (ng/mL)	41.76 ± 26.73	0.047 (−0.002–0.096)	0.061
5-Methyl-tetrahydrofolate (ng/mL)	39.49 ± 24.98	0.045 (−0.006–0.096)	0.086
BMI (kg/m²)	28.36 ± 7.25	3.945 (3.762–4.128)	<0.001

β: regression coefficient. CI: confidence interval. LAP: lipid accumulation product. HOMA-IR: homeostasis model assessment of insulin resistance. BMI: body mass index. ALT: alanine aminotransferase. AST: aspartate aminotransferase. LDH: lactate dehydrogenase. RBC: red blood cell. Statistical significance was defined as p<0.05.

### Serum cotinine in relation to LAP

Survey-weighted multivariable models were constructed to quantify how serum cotinine corresponded to LAP values (Table 3). After full covariate adjustment (Model 3), cotinine retained a positive regression coefficient for LAP (β=0.019; 95% CI: 0.011–0.028; p<0.001). When serum cotinine was categorized into tertiles, participants in the highest tertile exhibited significantly higher LAP values than those in the lowest tertile after full adjustment (β=3.142; 95% CI: 0.580–5.705; p=0.016). In addition, a significant positive trend across cotinine tertiles was observed after full adjustment (p for trend=0.017). Results from unadjusted and partially adjusted models are presented in Table 3.

**Table 3:** Survey-weighted association between serum cotinine and LAP among US NHANES 2013–2018 participants (N=6409)

Exposure	Model 1 β (95% CI)	p	Model 2 β (95% CI)	p	Model 3 β (95% CI)	p
Serum cotinine (ng/mL)	0.013 (0.001–0.025)	0.035	0.010 (−0.000–0.021)	0.056	0.019 (0.011–0.028)	<0.001
**Serum cotinine tertiles** (ng/mL)						
Q1 (0.011–0.015)	ref.		ref.		ref.	
Q2 (0.016–0.190)	-2.394 (−6.059–1.272)	0.201	-2.280 (−5.513–0.954)	0.167	-1.758 (−4.293–0.776)	0.174
Q3 (0.191–1030.0)	1.536 (−2.089–5.162)	0.406	0.589 (−2.641–3.819)	0.721	3.142 (0.580–5.705)	0.016
p for trend		0.424		0.736		0.017

Model 1 was unadjusted. Model 2 was adjusted for age, sex, and BMI. Model 3 was adjusted as for Model 2 plus ALT, albumin, AST, globulin, LDH, uric acid, RBC folate, serum total folate, and 5-methyl-tetrahydrofolate. β: regression coefficient. CI: confidence interval. LAP: lipid accumulation product. BMI: body mass index. ALT: alanine aminotransferase. AST: aspartate aminotransferase. LDH: lactate dehydrogenase. RBC: red blood cell. Statistical significance was defined as p<0.05.

### HOMA-IR in relation to LAP

A parallel set of survey-weighted regression models was applied to examine HOMA-IR with respect to LAP (Table 4). In Model 3, HOMA-IR was positively associated with LAP (β=1.345; 95% CI: 1.198–1.492; p<0.001). LAP increased across HOMA-IR tertiles, with significant trends in all models (p for trend <0.001). Results from unadjusted and partially adjusted models are presented in Table 4.

**Table 4:** Survey-weighted association between HOMA-IR and LAP among US NHANES 2013–2018 participants (N=6409)

Exposure	Model 1 β (95% CI)	p	Model 2 β (95% CI)	p	Model 3 β (95% CI)	p
HOMA-IR	2.482 (2.282–2.683)	<0.001	1.323 (1.132–1.514)	<0.001	1.345 (1.198–1.492)	<0.001
**HOMA-IR tertiles**						
Q1 (0.026–1.753)	ref.		ref.		ref.	
Q2 (1.754–3.375)	19.815 (16.480–23.151)	<0.001	8.982 (5.761–12.203)	<0.001	6.938 (4.430–9.446)	<0.001
Q3 (3.376–269.413)	58.041 (54.699–61.383)	<0.001	29.343 (25.633–33.053)	<0.001	25.611 (22.634–28.587)	<0.001
p for trend		<0.001		<0.001		<0.001

Model 1 was unadjusted. Model 2 was adjusted for age, sex, and BMI. Model 3 was adjusted as for Model 2 plus ALT, albumin, AST, globulin, LDH, uric acid, RBC folate, serum total folate, and 5-methyl-tetrahydrofolate. β: regression coefficient. CI: confidence interval. LAP: lipid accumulation product. BMI: body mass index. ALT: alanine aminotransferase. AST: aspartate aminotransferase. LDH: lactate dehydrogenase. RBC: red blood cell. Statistical significance was defined as p<0.05.

### Mediating role of HOMA-IR

The contribution of HOMA-IR to the cotinine–LAP association was further evaluated using mediation analysis based on Model 3 (Figure 2, Table 5). The overall association between cotinine and LAP was statistically significant (β=0.042; 95% CI: 0.008–0.043; p=0.004). After HOMA-IR was entered as the mediator, the direct effect of serum cotinine remained evident (β=0.039; 95% CI: 0.002–0.038; p=0.020). The pathway through HOMA-IR was also significant, with an indirect effect estimate of 0.003 (95% CI: 0.000–0.013; p=0.046). The proportion mediated by HOMA-IR was 7.4% (proportion mediated=0.074, p=0.050) (Table 5, Figure 2).

**Figure 2:**
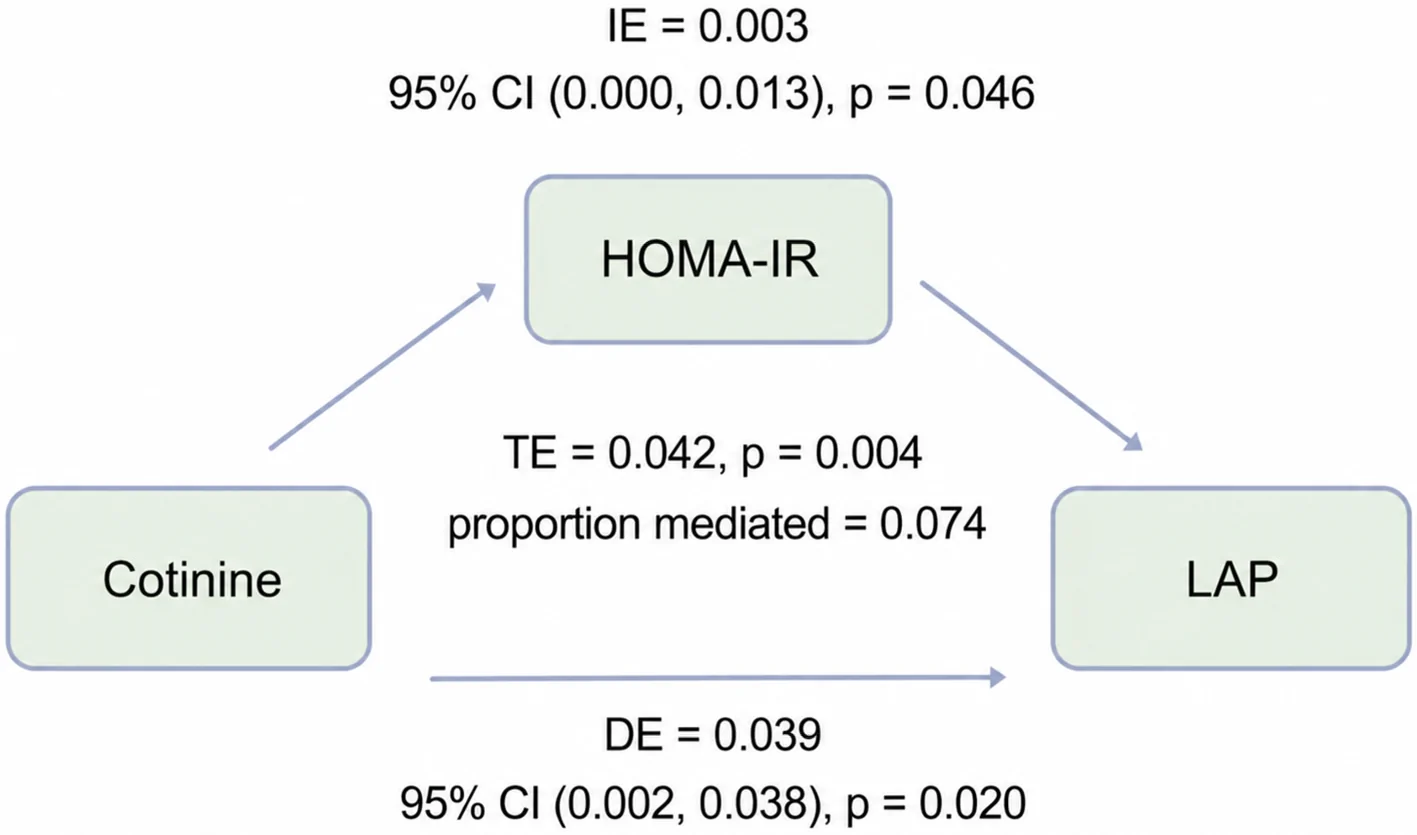
Statistical mediation model for the association between serum cotinine and LAP through HOMA-IR, among US NHANES 2013–2018 participants (N=6409)

**Table 5:** Statistical mediation analysis of HOMA-IR in the association between serum cotinine and LAP among US NHANES 2013–2018 participants (N=6409)

Effect	Estimate	95% CI Lower	95% CI Upper	p
Total effect	0.042	0.008	0.043	0.004
Mediation effect	0.003	0.000	0.013	0.046
Direct effect	0.039	0.002	0.038	0.020
Proportion mediated	0.074	0.004	0.778	0.050

CI: confidence interval. HOMA-IR: homeostasis model assessment of insulin resistance. LAP: lipid accumulation product. Because of the cross-sectional design, the mediation results should be interpreted as statistical decomposition rather than evidence of causal mediation. Statistical significance was defined as p<0.05.

## Discussion

Using a weighted sample from NHANES 2013–2018 with complete data on serum cotinine, LAP, HOMA-IR, and covariates, this study identified a positive association between serum cotinine and LAP after multivariable adjustment. Participants with higher LAP levels also exhibited elevated cotinine and HOMA-IR levels. Mediation analysis further indicated that HOMA-IR explained only a modest proportion of the cotinine–LAP association. Overall, higher serum cotinine, reflecting greater recent nicotine intake, was associated with higher LAP, while HOMA-IR explained only a small proportion of this association.

These findings are broadly consistent with previous studies linking smoking or tobacco smoke exposure to dyslipidemia and unfavorable fat distribution. Using cotinine as a biomarker of recent nicotine exposure, an NHANES analysis reported a less favorable lipid profile among smokers, primarily reflected by higher triglyceride levels, lower high-density lipoprotein cholesterol (HDL-C), and an increased prevalence of related lipid abnormalities^26^. The CARDIA cohort study found that, although current smokers had a lower BMI than never smokers, they exhibited greater abdominal intermuscular adipose tissue deposition and a higher visceral-to-subcutaneous fat ratio, along with poorer muscle quality. Higher baseline cotinine levels were also associated with an increased visceral-to-subcutaneous fat ratio at follow-up^27^. Another study in an Asian population reported that individuals with greater cumulative smoking exposure tended to have a less favorable abdominal fat ratio and a larger visceral-to-subcutaneous adipose tissue area ratio, even after adjusting for BMI^28^. Taken together, these studies suggest that smoking-related metabolic alterations may not be fully captured by changes in overall body weight, but may involve alterations in fat distribution, dyslipidemia, and ectopic fat deposition. The observed association between serum cotinine and LAP may therefore indicate a relationship between recent nicotine intake and a composite metabolic phenotype involving abdominal lipid burden and triglyceride dysregulation. Nevertheless, cotinine-based evidence across lipid-related outcomes has not been completely uniform. A clinic-based study among Turkish immigrants with type 2 diabetes reported that increasing serum cotinine quartiles were not significantly associated with lipid-related indices^16^. This difference may be partly explained by variations in study population, sample size, exposure categorization, and outcome definition, because that study focused on conventional lipid-related indices rather than LAP^16^.

From a public health perspective, this finding may have practical implications. A normal BMI does not necessarily indicate low metabolic risk; previous studies have shown that individuals with normal body weight but central obesity may still face a higher risk of cardiovascular mortality or a greater burden of cardiometabolic risk factors^29,30^. Because BMI provides limited information on fat distribution or lipid-related metabolic burden, LAP may offer additional value in identifying hidden cardiometabolic risk^4^. The cotinine-LAP association observed in this study suggests that individuals with higher serum cotinine levels may have higher LAP even in the absence of marked general obesity. Therefore, metabolic risk assessment may benefit from considering not only BMI but also objective biomarkers of recent nicotine intake and indicators of abdominal lipid accumulation.

Mechanistically, the association between serum cotinine and LAP may be consistent with pathways involving nicotine-related adipose tissue dysfunction, enhanced lipid mobilization, and increased triglyceride metabolic burden. Cotinine should be interpreted primarily as a biomarker of recent nicotine intake rather than as a direct causal factor^13,14^. As a composite index of lipid accumulation, LAP may increase when abdominal adiposity, triglyceride burden, or both are elevated^4^. Previous studies have suggested that smoking may affect adipocyte function, inflammatory responses, adipokine expression, and lipid storage capacity, thereby impairing adipose tissue metabolic homeostasis^31,32^. In addition, systemic nicotine exposure may stimulate adipose tissue lipolysis through cholinergic and catecholaminergic pathways, thereby increasing free fatty acid delivery to the liver^33^. This process may promote hepatic triglyceride storage and very-low-density lipoprotein (VLDL) secretion, which could increase the triglyceride component of LAP^19^. Therefore, nicotine-related lipid mobilization and hepatic triglyceride metabolism may partly explain the observed association between higher serum cotinine and higher LAP^4,19,33^.

The partial mediating role of HOMA-IR also has biological plausibility. Nicotine can affect multiple central and peripheral aspects of glucose metabolic regulation, including interference with insulin sensitivity, alterations in islet hormone secretion, and changes in glucose metabolism in skeletal muscle and the liver^18^. Under insulin-resistant conditions, increased fatty acid delivery to the liver can promote hepatic triglyceride accumulation and the production of triglyceride-rich VLDL, thereby contributing to hypertriglyceridemia and obesity-related dyslipidemia^19^. Because LAP includes both waist circumference and triglycerides, insulin resistance may influence LAP either by affecting abdominal lipid accumulation (reflected by waist circumference) or by promoting hypertriglyceridemia (affecting its biochemical component)^4,19^.

Although HOMA-IR statistically mediated part of the association between serum cotinine and LAP, the mediated proportion was only 7.4%, indicating that most of the association remained unexplained by insulin resistance as captured by HOMA-IR. Several alternative or complementary pathways may contribute to the remaining association. Recent nicotine intake may be related to LAP through insulin-resistance-independent lipid mobilization. Nicotine can stimulate sympathetic activity and catecholamine-related pathways, thereby enhancing adipose tissue lipolysis and increasing free fatty acid flux to the liver^33^. Increased fatty acid delivery may promote hepatic triglyceride synthesis and VLDL secretion, which could increase the triglyceride component of LAP^4,19^. In addition, smoking-related adipose tissue dysfunction may alter adipocyte differentiation, lipid storage capacity, adipokine secretion, and local inflammatory responses, leading to abnormal fat distribution and ectopic lipid deposition^31,32^. These changes may affect waist circumference and triglyceride levels even when HOMA-IR does not fully capture the metabolic disturbance. Oxidative stress and low-grade inflammation related to nicotine exposure may contribute to dysregulated lipid metabolism and adipose tissue remodeling through pathways not completely reflected by fasting glucose and insulin concentrations^10,31,32^. In addition to these biological pathways, residual confounding should also be considered. Behavioral and socioeconomic factors, such as diet, alcohol consumption, physical activity, medication use, smoking intensity, cumulative smoking history, and nicotine source, may influence serum cotinine levels, LAP, or both. Therefore, the remaining association should be interpreted as reflecting a combination of biological pathways and residual confounding rather than a pathway fully explained by insulin resistance.

### Limitations

Several methodological constraints should be considered. First, the cross-sectional design of NHANES precludes causal inference regarding the temporal sequence among serum cotinine, HOMA-IR, and LAP. Second, serum cotinine, HOMA-IR, and LAP were measured at a single time point. Serum cotinine reflects recent nicotine exposure and may not capture long-term smoking history, cumulative exposure, temporal variation in exposure, or whether the exposure came from combustible cigarettes, e-cigarettes, nicotine replacement therapy, or secondhand smoke. Similarly, single measurements of HOMA-IR and LAP may not fully represent long-term insulin resistance or lipid accumulation status. Such within-person variability may lead to exposure or outcome misclassification and could attenuate or otherwise influence the observed associations. Third, LAP, while integrating waist circumference and triglycerides, remains a surrogate index and is not equivalent to imaging-based measures of visceral or ectopic fat. Fourth, HOMA-IR is a widely used but indirect marker of insulin resistance. Fifth, although we adjusted for multiple covariates, residual confounding may still exist. Notably, race/ethnicity and education were not included in the primary models, despite their potential associations with smoking behavior, nicotine exposure patterns, cotinine metabolism, diet, socioeconomic status, healthcare access, and cardiometabolic risk. Consequently, incomplete adjustment for these and related social or behavioral factors may have biased the estimated association between serum cotinine and LAP. Finally, the mediation analysis was based on cross-sectional data. Therefore, the assumed temporal sequence from serum cotinine to HOMA-IR and then to LAP cannot be established. The indirect effect should not be interpreted as evidence of causal mediation, but rather as a statistical decomposition of the observed association. Reverse causation or shared underlying metabolic processes may also contribute to the observed relationships. In addition, HOMA-IR accounted for only a small proportion of the cotinine–LAP association, so the mediation findings should be considered exploratory. Prospective studies with repeated exposure assessment, imaging-based fat assessment, inflammatory markers, adipokines, and metabolomic indicators are warranted to elucidate the underlying mechanisms.

## Conclusions

In the NHANES 2013–2018 population, higher serum cotinine, a biomarker of recent nicotine intake, was positively associated with LAP in cross-sectional analyses. HOMA-IR statistically explained only a small proportion of this association. Given the cross-sectional design, these findings should be interpreted as associations rather than evidence of temporal or causal relationships. Prospective studies and mechanistic investigations are needed to clarify the relationships among nicotine intake, insulin resistance, and abdominal lipid accumulation.

## References

[1] Powell-Wiley TM, Poirier P, Burke LE (2021). Obesity and cardiovascular disease: a scientific statement from the American heart association. Circulation.

[2] Carr MC, Brunzell JD (2004). Abdominal obesity and dyslipidemia in the metabolic syndrome: importance of type 2 diabetes and familial combined hyperlipidemia in coronary artery disease risk. J Clin Endocrinol Metab.

[3] Nuttall FQ (2015). Body mass index: obesity, BMI, and health: a critical review. Nutr Today.

[4] Kahn HS (2005). The “lipid accumulation product” performs better than the body mass index for recognizing cardiovascular risk: a population-based comparison. BMC Cardiovasc Disord.

[5] Kyrou I, Panagiotakos DB, Kouli GM (2018). Lipid accumulation product in relation to 10-year cardiovascular disease incidence in Caucasian adults: the ATTICA study. Atherosclerosis.

[6] Witarto BS, Witarto AP, Visuddho V (2024). Gender-specific accuracy of lipid accumulation product index for the screening of metabolic syndrome in general adults: a meta-analysis and comparative analysis with other adiposity indicators. Lipids Health Dis.

[7] Yang SH, Yoon J, Lee YJ, Park B, Jung DH (2022). Lipid accumulation product index predicts new-onset type 2 diabetes among non-obese Koreans: a 12-year longitudinal study. Diabetes Metab Syndr Obes.

[8] Dai H, Wang W, Chen R, Chen Z, Lu Y (2017). Lipid accumulation product is a powerful tool to predict non-alcoholic fatty liver disease in Chinese adults. Nutr Metab (Lond).

[9] GBD 2015 Tobacco Collaborators (2017). Smoking prevalence and attributable disease burden in 195 countries and territories, 1990–2015: a systematic analysis from the Global Burden of Disease Study 2015. The Lancet.

[10] Rehman K, Haider K, Akash MSH (2021). Cigarette smoking and nicotine exposure contributes for aberrant insulin signaling and cardiometabolic disorders. Eur J Pharmacol.

[11] Sun K, Liu J, Ning G (2012). Active smoking and risk of metabolic syndrome: a meta-analysis of prospective studies. PLoS One.

[12] Pan A, Wang Y, Talaei M, Hu FB, Wu T (2015). Relation of active, passive, and quitting smoking with incident type 2 diabetes: a systematic review and meta-analysis. Lancet Diabetes Endocrinol.

[13] Benowitz NL, Hukkanen J, Jacob P (2009). Nicotine chemistry, metabolism, kinetics and biomarkers. Handb Exp Pharmacol.

[14] Benowitz NL, Bernert JT, Foulds J (2020). Biochemical verification of tobacco use and abstinence: 2019 update. Nicotine Tob Res.

[15] Jeong SH, Jang BN, Kim SH, Jang SI, Park EC (2021). Investigation of the association between smoking behavior and metabolic syndrome using lipid accumulation product index among South Korean adults. Int J Environ Res Public Health.

[16] Sukhram SD, Zarini GG, Shaban LH, Vaccaro JA, Sukhram AR (2023). Serum cotinine as a predictor of lipid-related indices in Turkish immigrants with type 2 diabetes: a clinic-based cross-sectional study. Front Med (Lausanne).

[17] Hou X, Gao Z, Sun T, Dong X, Xu L (2025). Examining the associations among adults’ physical activity, serum cotinine, and the risk of metabolic syndrome. BMC Public Health.

[18] Chen Z, Liu XA, Kenny PJ (2023). Central and peripheral actions of nicotine that influence blood glucose homeostasis and the development of diabetes. Pharmacol Res.

[19] Klop B, Elte JW, Cabezas MC (2013). Dyslipidemia in obesity: mechanisms and potential targets. Nutrients.

[20] Matthews DR, Hosker JP, Rudenski AS, Naylor BA, Treacher DF (1985). Homeostasis model assessment: insulin resistance and beta-cell function from fasting plasma glucose and insulin concentrations in man. Diabetologia.

[21] Mirmiran P, Bahadoran Z, Azizi F (2014). Lipid accumulation product is associated with insulin resistance, lipid peroxidation, and systemic inflammation in type 2 diabetic patients. Endocrinol Metab (Seoul).

[22] Mazidi M, Kengne AP, Katsiki N, Mikhailidis DP, Banach M (2018). Lipid accumulation product and triglycerides/glucose index are useful predictors of insulin resistance. J Diabetes Complications.

[23] Li J, Wang D, Tian H, Yang J, Xia H (2024). Association between lipid accumulation products and stress urinary incontinence: a cross-sectional study from NHANES 2005 to 2018. Lipids Health Dis.

[24] (2026). Centers for Disease Control and Prevention, National Center for Health Statistics. National Health and Nutrition Examination Survey: Laboratory Methods.

[25] Tingley D, Yamamoto T, Hirose K, Keele L, Imai K (2014). Mediation: R package for causal mediation analysis. J Stat Softw.

[26] Jain RB, Ducatman A (2018). Associations between smoking and lipid/lipoprotein concentrations among US adults aged ≥20 years. J Circ Biomark.

[27] Terry JG, Hartley KG, Steffen LM (2020). Association of smoking with abdominal adipose deposition and muscle composition in Coronary Artery Risk Development in Young Adults (CARDIA) participants at mid-life: a population-based cohort study. PLoS Med.

[28] Fujiyoshi A, Miura K, Kadowaki S (2016). Lifetime cigarette smoking is associated with abdominal obesity in a community-based sample of Japanese men: the Shiga Epidemiological Study of Subclinical Atherosclerosis (SESSA). Prev Med Rep.

[29] Sahakyan KR, Somers VK, Rodriguez-Escudero JP (2015). Normal-weight central obesity: implications for total and cardiovascular mortality. Ann Intern Med.

[30] Shirasawa T, Ochiai H, Yoshimoto T (2019). Associations between normal weight central obesity and cardiovascular disease risk factors in Japanese middle-aged adults: a cross-sectional study. J Health Popul Nutr.

[31] Kos K (2020). Cardiometabolic morbidity and mortality with smoking cessation, review of recommendations for people with diabetes and obesity. Curr Diab Rep.

[32] Wang Z, Wang D, Wang Y (2017). Cigarette smoking and adipose tissue: the emerging role in progression of atherosclerosis. Mediators Inflamm.

[33] Andersson K, Arner P (2001). Systemic nicotine stimulates human adipose tissue lipolysis through local cholinergic and catecholaminergic receptors. Int J Obes Relat Metab Disord.

